# The AI4GH community of practice: strengthening LMIC-Led artificial intelligence for global health

**DOI:** 10.1038/s41746-026-02748-6

**Published:** 2026-05-21

**Authors:** Mylena Maria Guedes de Almeida, Mercedes Rumi, Marina Bowder, Prince Adjei, Bryain Maradiaga-Mendoza, Marina Albada, Adam Dale, Salvia Zeeshan, Simran Siraj, Nour El Arnaout, Rosalind Parkes-Ratanshi, Anneka Wickramanayake, Peiling Yap, Cintia Cejas, Trudie Lang

**Affiliations:** 1 Visiting Student, Nuffield Department of Medicine, University of Oxford, Oxford, UK; 2 Escola Paulista de Medicina, Universidade Federal de São Paulo, São Paulo, Brazil; 3 The Global Health Network, University of Oxford, Oxford, UK; 4 Global Health and Infectious Diseases Group, Kumasi Centre for Collaborative Research in Tropical Medicine, Kwame Nkrumah University of Science and Technology, Kumasi, Ghana; 5 Department of Computer Engineering, Kwame Nkrumah University of Science and Technology, Kumasi, Ghana; 6 Global One Health Research Group, Bernhard Nocht Institute of Tropical Medicine, Hamburg, Germany; 7 PHC Global, Karachi, Pakistan; 8 Global Health Institute, American University of Beirut, Beirut, Lebanon; 9 School of Medicine, Dentistry and Biomedical Sciences, Queen’s University Belfast, Belfast, UK; 10 Infectious Diseases Institute, Makerere University, Kampala, Uganda; 11 Jacaranda Health, Nairobi, Kenya; 12 HealthAI - The Global Agency for Responsible AI in Health, Geneva, Switzerland; 13 Instituto de Efectividad Clínica y Sanitaria, Buenos Aires, Argentina; 14 Centre for Tropical Medicine and Global Health, Nuffield Department of Medicine, University of Oxford, Oxford, UK

**Keywords:** Business and industry, Health care, Mathematics and computing, Scientific community, Social sciences

## Abstract

Efforts to research, implement and scale responsible Artificial Intelligence (AI) for addressing health challenges in low- and middle-income countries (LMICs) are often fragmented. It limits collaboration and slows progress. Communities of Practice (CoPs) offer a promising approach to fostering shared learning and supporting the use of AI to strengthen health systems and improve health outcomes. The Artificial Intelligence for Global Health Community of Practice (AI4GH CoP), established in 2023, connects researchers, implementers and policymakers across the Global South to co-create and scale responsible AI solutions for public health priorities. This case study examines the design, functioning and outputs of the AI4GH CoP using a structured community roadmap. It illustrates a model for advancing responsible AI in LMICs by reinforcing local expertise and facilitating context-specific knowledge exchange and capacity building in ethical, regulatory and operational aspects of AI in global health. These lessons can inform the establishment of similar CoPs.

## Introduction

From surveillance to diagnostics and clinical care, artificial intelligence (AI) is rapidly transforming the global health landscape. It holds significant promise for strengthening health systems and reducing inequalities^1–3^. However, the underrepresentation of low- and middle-income countries (LMICs) in AI development and implementation research limits the extent to which AI can achieve this impact. With AI solutions concentrated in high-income settings, LMICs risk being relegated to the role of data providers rather than decision-makers^4,5^. This imbalance has been described as a form of data colonialism, in which data from underrepresented populations may be gathered without equitable participation, control or benefit^5^. Such dynamics can deepen existing health and North-South divides.

Responding to these complex issues calls for the development of responsible AI. This term refers to the design and implementation of systems that are ethical, inclusive, secure and human-centred, while protecting privacy and respecting human rights^6^. Achieving responsible AI and stronger representation of LMICs requires empowering local actors and fostering collaboration among stakeholders^1^. However, researchers and implementers often face limited incentives and lack spaces to share experiences, exchange lessons, form partnerships and consolidate collaborations. As a result, efforts are often fragmented and confined to individual institutions or projects^7^. This disconnect reduces opportunities for collective learning and slows progress toward AI solutions that reflect local priorities, integrate into health systems and deliver sustainable impact in LMICs^8^.

To bridge this fragmentation and facilitate shared learning, Communities of Practice (CoPs) present a promising approach. Originating from Wenger’s work, CoPs are defined as ‘groups of people who share a concern or a passion for something they do and learn how to do it better as they interact regularly’^9^. First described in business as a mechanism for sharing tacit knowledge^10^, CoPs have been recognised as particularly relevant in healthcare^11^. They have been used to promote peer learning, foster collaboration across institutions and strengthen research capacity through knowledge transfer^12,13^. These features make CoPs well-suited to the emerging field of AI, where rapid innovation and ethical concerns demand constant multidisciplinary dialogue and knowledge exchanges.

To explore this potential, the Artificial Intelligence for Global Health (AI4GH) CoP was launched in 2023. It has operated on a dedicated virtual platform^14^, which serves as a knowledge hub. This CoP is part of the larger AI4GH initiative^15^, funded by Canada’s International Development Research Centre (IDRC) and the UK Foreign, Commonwealth & Development Office (FCDO). The initiative has supported interdisciplinary research projects across sub-Saharan Africa, Asia, Latin America and the Caribbean (LAC) and the Middle East and North Africa (MENA). These projects focus on AI applications in Sexual, Reproductive and Maternal Health (SRMH) and Epidemic/Pandemic Prevention, Preparedness and Response (E/PPPR). The CoP’s goal is to connect researchers, implementers and decision-makers based in LMICs, both inside and outside the AI4GH initiative, to maximise the impact of investments and support equitable, responsible AI in health.

The literature frequently highlights the importance of cross-sector collaboration in facilitating knowledge sharing and advancing best practices in AI for health^16–18^, but documented examples of such partnerships remain limited. Although various organisations and collaborations address AI in healthcare, the ways they operate, how they are characterised, their potential functioning as CoPs, and their variation across contexts and resource settings remain underexplored^19^. Therefore, in this case study, we aim to describe and reflect on the design, activities and outputs of the AI4GH CoP, using a community roadmap to guide the report and to identify strengths and gaps. The purpose of this process is to support the ongoing development of the AI4GH CoP and provide transferable insights for building equitable AI-for-health research ecosystems in LMICs.

## Results

Table 1 presents responses to the 27 Community Roadmap^20^ questions. These responses were derived from CoP documentary sources and refined through iterative member review following the process detailed in the ‘Methods’ section. The questions guided the description of the AI4GH CoP’s current status across eight domains: Vision, Governance, Leadership, Convening, Collaboration/Cooperation, Community Management, User Experience and Measurement.

**Table 1:** AI4GH community of practice roadmap

AI4GH community of practice roadmap	
Vision	
(1) What is the challenge you want to address/the problem to solve?	Gaps in research capacity, collaboration and knowledge transfer limiting LMICs in developing, implementing and scaling responsible AI in healthcare.
(2) What is your long-term goal?	To strengthen healthcare systems and reduce regional health inequities through responsible AI solutions that improve SRMH outcomes and support effective E/PPPR.
(3) Community purpose: What is the community’s *raison d’être*^a^ in support of the vision?	To connect LMIC-based researchers, implementers and decision-makers in a global Community of Practice that fosters knowledge sharing, co-creation and dissemination of responsible AI solutions for SRMH and E/PPPR.
(4) Objectives: What is your strategy to reach your community vision?	Use the AI4GH Knowledge Hub as a central, multilingual platform for knowledge sharing and translation, supported by capacity-building activities and thematic/cross-cutting working groups.
Governance	
(5) How do you work together, take decisions and act on them?	Decisions and actions are coordinated through a Steering Committee and thematic working groups. Stakeholders are collaboratively developing the Terms of Reference (ToRs).
(6) Stakeholder mapping: Define your membership and the surrounding community ecosystem. Who are the actors involved in/impacted by the community?	**• Internal actors:** AI4GH-funded research teams, funders, implementation partners, The Global Health Network core group **• External actors:** Policymakers, practitioners, target beneficiary group(s) and potentially their family members, communities, public institutions, private sector, researchers and everyone involved in the research process.
(7) Risk-free environment: What are key elements building trust and guaranteeing a safe place?	Inclusive regional representation (Africa, Asia, MENA, LAC), cross-sector working groups, transparent oversight via the Steering Committee, clear ToRs and a culture of shared learning and mutual support/respect.
Leadership	
(8) How will you ensure strong leadership participation?	Consortium leading knowledge sharing and translation efforts and facilitating distributed leadership among regional partners, with strong support from funders.
(9) Core group: How do you get your core group to steer the community?	The core group at The Global Health Network (Project Manager, MEL Manager, Regional Coordinators for LAC, Africa and Asia) leads the knowledge hub, coordinates activities and facilitates knowledge translation among AI4GH research teams and the broader community. Regional coordinators for Asia, Africa and Latin America support regional partnerships and priorities.
(10) Investment and sponsorship: What support do you need from management? How do you get them involved and create participation opportunities?	Funders provide ongoing support, feedback and strategic guidance through the Steering Committee and consultations, helping prioritise efforts and align activities with community needs and strategic goals.
Convening	
(11) What kind of convening opportunities/events fit with your community in general?	Online and face-to-face, regional and global events, including workshops, webinars and meetings tailored to AI in global health. There are regular meetings with AI4GH partners, all-partner meetings and individual meetings of the core group with each partner.
(12) Communication, connection and conversation: What convening opportunities will you design to create and encourage connections, conversations and communication?	**•** Quarterly Steering Committee meetings. **•** Working group meetings. **•** Annual in-person all-partner meeting. **•** Hybrid regional workshops (LAC, Africa, Asia, MENA). **•** Webinars. **•** Knowledge Hub events page^22^. **•** Shared event calendar. **•** Newsletter with updates, outcomes, experiences and good practices^36^.
(13) Boundary-spanning: How do you regularly feed your community with external expertise and promote access to other networks?	**•** Open webinars. **•** Global conference presentations. **•** Knowledge Hub’s ‘Connect and Collaborate’ feature, creating opportunities to engage with the wider AI in health community^37^. **•** The ‘Contact us’ and ‘Would you like to be assigned a DOI?’ features, which encourage queries, suggestions and contributions^38^. **•** Connection with other The Global Health Network knowledge hubs^39^. **•** Interactive map showcasing all projects^21^.
Collaboration/cooperation	
(14) How do you make members collaborate and/or cooperate to enrich the common practice and produce knowledge assets/qualitative deliverables?	Thematic/cross-cutting working group meetings which facilitate collaboration and cooperation.
(15) Coordination: How do you coordinate members’ work towards delivering on the objectives agreed?	The Global Health Network team organises meetings, facilitates agendas with AI4GH partners, documents discussions, tracks action items and reports progress and challenges to funders.
(16) Co-creation: What content needs to be curated/synthesised/ co-created and what methods will you use to succeed in this?	Curate/synthesise ethical AI guidelines, training toolkits, impact frameworks and region-specific resources through scoping activity and gap analysis^40^. Curate Responsible AI resources^41^.
Community management	
(17) What role and tasks will the community manager perform?	Maintain/update the Knowledge Hub, facilitate connections, coordinate content contributions, manage communications (newsletters, social media), organise convenings and track metrics.
(18) In real life and online: How will you combine and ensure the flow between real-life and online, asynchronous and synchronous community interactions?	Blend in-person workshops/meetings with online meetings and tools (knowledge hub, webinars, shared documents) for synchronous and asynchronous participation.
(19) Facilitation: What facilitation methods do you need to get the best out of the community dynamic social process?	Encourage regional coordination, iterative feedback loops through the Knowledge Hub, regional hubs, thematic/cross-cutting working groups and regular meetings with different groups of stakeholders.
User experience	
(20) How to ensure a user centric experience for the tasks (you want) members (want) to do in the community?	Designing hub tools based on member feedback, providing multilingual content, ensuring open access, assigning DOI for resources shared.
(21) Experience design: What are the community’s personas and their user requirements, as well as the pain points to address?	LMIC researchers need ethical AI guidance, training resources and networking opportunities. Pain points: lack of national AI policies and regulations, limited datasets.
(22) Support: What process and content do you need to put in place to provide support?	Resource bank, toolkits, guidelines, e-learning modules, event promotion, support from partners for content sharing.
Measurement	
(23) What have you achieved?	Expanded Knowledge Hub membership and visibility; delivered workshops; expanded resource bank; established governance structure; created working groups; grew newsletter subscribers.
(24) What can you learn from those measurements and how will you address the challenges/obstacles?	Measuring short-term indicators has demonstrated their value for tracking activity but also their limitation in capturing broader impact. As the CoP evolves, greater insight can be gained by incorporating focus group interviews and other methods to assess medium- and long-term outcomes, including changes in practice and policy.
(25) Vitality: What habits and behaviours should you observe and encourage?	Regular contributions to hub, active working group participation, meeting attendance, resource sharing, cross-region collaborations.
(26) Results: How do you measure the key results in delivering on the community objectives?	Track Knowledge hub website metrics, event participation and collect impact stories from AI4GH-funded projects.
(27) How will you capture impact stories?	Impact stories project: Interviews with each project PI’s or AI4GH research teams, encouraging reflection on challenges, learning and capacity built. The interviews are converted into impact stories for different media formats: pdf, carousel style and videos to be published in the hub and shared on social media.

^a^*raison d'être*: the most important reason for somebody’s or something’s existence; *AI* Artificial Intelligence, *AI4GH* Artificial Intelligence for Global Health, *E/PPPR* Epidemic/Pandemic Prevention, Preparedness and Response, *LAC* Latin America and the Caribbean, *LMICs* low- and middle-income countries, *MENA* Middle East and North Africa, *PI* Principal Investigator, *SRMH* Sexual, Reproductive and Maternal Health, *ToRs* Terms of Reference. Community Roadmap questions from the European Union (2021)^20^, reused under the Creative Commons Attribution 4.0 International (CC BY 4.0) licence (https://creativecommons.org/licenses/by/4.0).

**Fig. 1:**
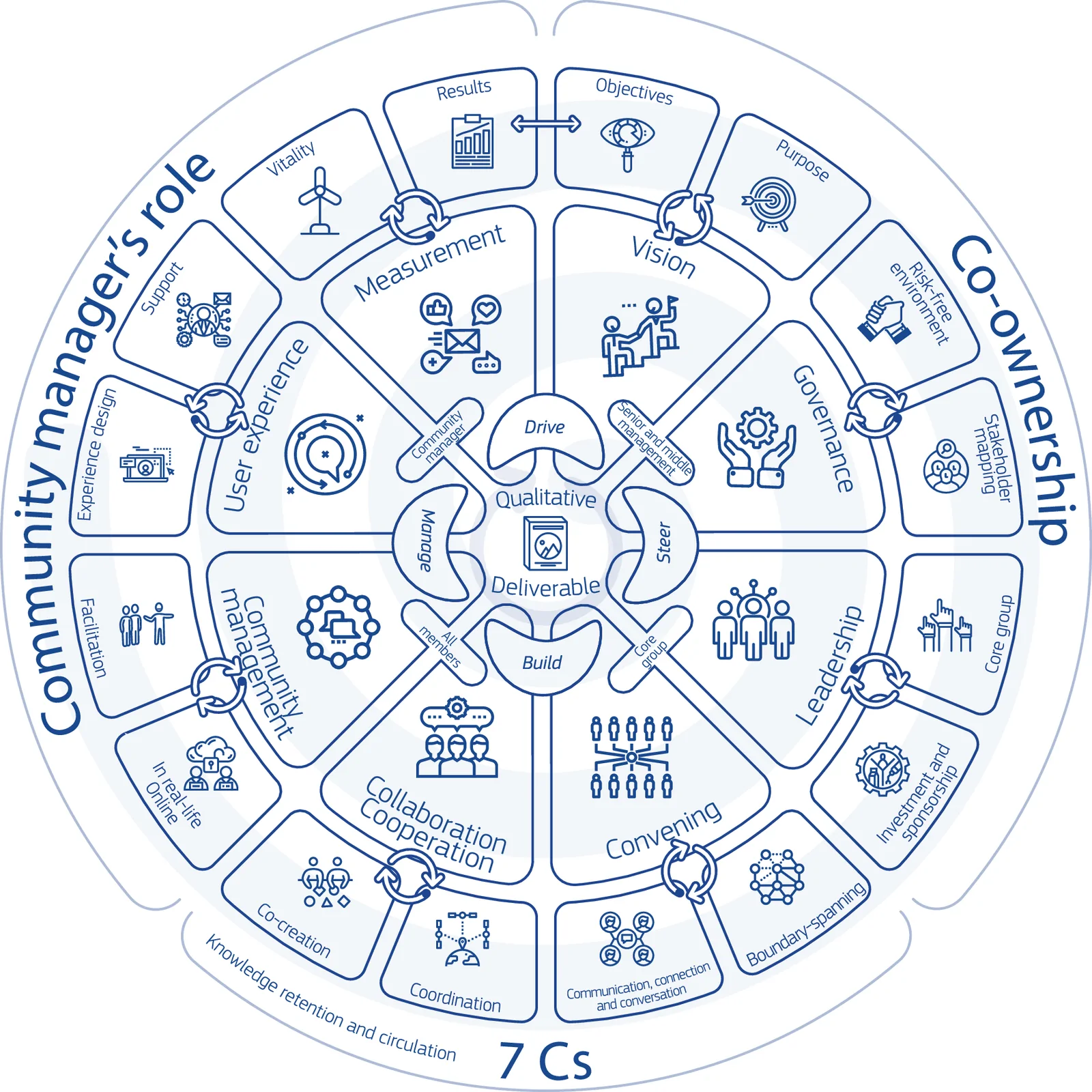
Communities of practice success wheel. © European Union, 2021^20^. Reused under the Creative Commons Attribution 4.0 International (CC BY 4.0) license (https://creativecommons.org/licenses/by/4.0).

The Community Roadmap questions also informed the assessment and learning process for each domain. For this analysis, we grouped the domains into three interconnected categories based on the community of practice success wheel^20^ (Fig. 1) and included an additional category that presents lessons emerging from the community assessment.

The Results are presented in four parts: (1) ‘Co-ownership’; (2) ‘Knowledge retention and circulation’; (3) ‘Community manager’s role’; and (4) ‘Overall assessment’. ‘Co-ownership’ comprises vision, governance and leadership. ‘Knowledge retention and circulation’ includes Convening, Collaboration, and Cooperation. ‘Community manager’s role’ encompasses Community Management, User Experience, and Measurement. ‘Overall assessment’ focuses on internal and transferable lessons from the community assessment.

### Co-ownership: vision, governance and leadership

The three co-ownership facets comprise participatory decision-making culture and community governance. After applying the Community Roadmap, we evaluated these facets as strong and internally well-defined. AI4GH CoP governance structure evolved to promote inclusive representation across regions and to facilitate cross-sector collaboration through working groups (WGs). The CoP benefits from a distributed leadership model, with regional partners and from the consortium leadership with strong support from sponsors. Table 2 presents an overview of the AI4GH initiative partners. Oversight and accountability are provided by the Steering Committee, composed of representatives from these partners. The Committee meets regularly to provide strategic guidance and review operational progress.

**Table 2:** AI4GH initiative partners

Partner, country	Project name	Regional focus
Funding partners		
The International Development Research Centre (IDRC), Canada Foreign, Commonwealth & Development Office (FCDO), United Kingdom	Artificial Intelligence for Global Health (AI4GH)^15^, part of Artificial Intelligence for Development (AI4D)^42^	Global South
Collaborator institutes		
The Global Health Network, United Kingdom	Artificial Intelligence for Global Health Research hub^14,43^	Global South
HealthAI-The Global Agency for Responsible AI in Health, Switzerland	Supporting Responsible AI for Global Health^44^	Global South
Sexual reproductive maternal health (SRMH) partners		
Institute for Clinical Effectiveness and Health Policy (IECS), Argentina	Centro de Inteligencia Artificial y Salud para América Latina y el Caribe (CLIAS)^45^	LAC
PHC Global, Pakistan GTA Foundation, Nepal	AI-Sarosh^46^	Asia
Global Health Institute, American University of Beirut (AUB), Lebanon	The Global Health and Artificial Intelligence Network in the Middle East and North Africa region (GHAIN MENA)^47^	MENA
Infectious Diseases Institute (IDI), Uganda Sunbird AI, Uganda Makerere University College of Computing and Information Science (COCIS), Uganda	Hub for Artificial Intelligence in Maternal Sexual and Reproductive Health (HASH)^48^	Africa
Jacaranda Health, Kenya	PROMPTS^49^	Africa
Epidemic/pandemic prevention, preparedness and response (E/PPPR) partner		
York University, Canada University of Toronto, Canada	AI4PEP^50^	Global South
Commercialisation partners		
Villgro Africa, Kenya	AI4H Africa^51^	Africa
Villgro Philippines, Philippines	AI4Health Asia^52^	Asia
Tecnológico de Monterrey, Mexico	PASIA^53^	LAC

*LAC* Latin America and the Caribbean, *MENA* Middle East and North Africa.

Partners in SRMH, E/PPPR and Commercialisation are grantees of the AI4GH initiative. Each grantee supports subgrantees: research teams working on AI projects. Despite their different thematic areas, all AI4GH-funded projects aim to promote ethical solutions, advance gender equality and inclusion and incorporate plans for sustainability, scalability, and impact.

Echoing this shared goal, the community assessment highlighted inclusion, gender equity and community engagement as central elements of the CoP vision, which is reflected in several initiatives. For example, projects within the AI4PEP initiative, which focuses on disease outbreak prevention, serve diverse populations, including residents of urban informal settlements and refugee camps. Among the SRMH partners, teams are developing AI chatbots in local languages, such as Quechua in Peru and Swahili in Kenya. The regional hub CLIAS in LAC focuses specifically on applying AI to improve health outcomes for vulnerable populations, including rural, disabled, Indigenous and Afro-descendant communities. Across Africa, projects are addressing SRMH priorities, enhancing tuberculosis diagnostics for people living with HIV, and developing other locally relevant applications of AI in healthcare. In the MENA and Asia regions, teams are tackling issues such as gender-based violence and maternal mental health.

These are only a few examples among a wide range of ongoing initiatives. Projects are co-created with communities, who contribute to solution development, testing and feedback. An overview of projects and their regional distribution is available on the CoP digital platform^21^.

**Fig. 2:**
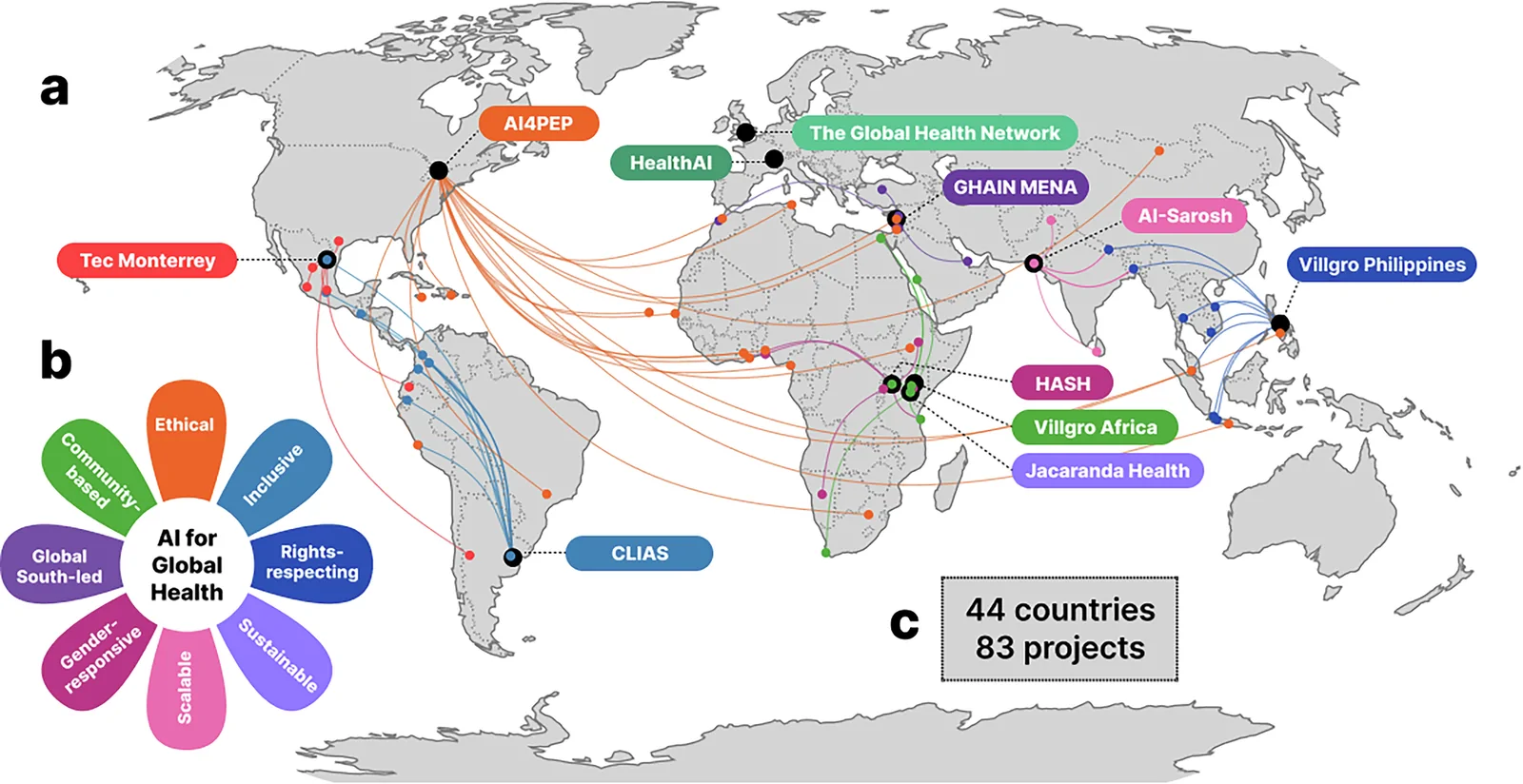
Overview of the AI4GH initiative. **a** Global and regional networks: larger black dots represent partners (grantees), and smaller dots represent AI projects (subgrantees). Lines connect subgrantees to their grantees. **b** All partners and projects are linked through the guiding principles of the AI4GH initiative. **c** Total counts of countries and projects. Colours are illustrative.

Figure 2 illustrates the regional and global networks, along with the guiding attributes for both the AI projects and the CoP as a whole: ethical, inclusive, rights-respecting, sustainable, scalable, gender-responsive, global south-led and community-based. This orientation reflects principles of equitable implementation, underpinning the initiative from evidence generation to knowledge translation.

### Knowledge retention and circulation: convening and collaboration/cooperation

**Fig. 3:**
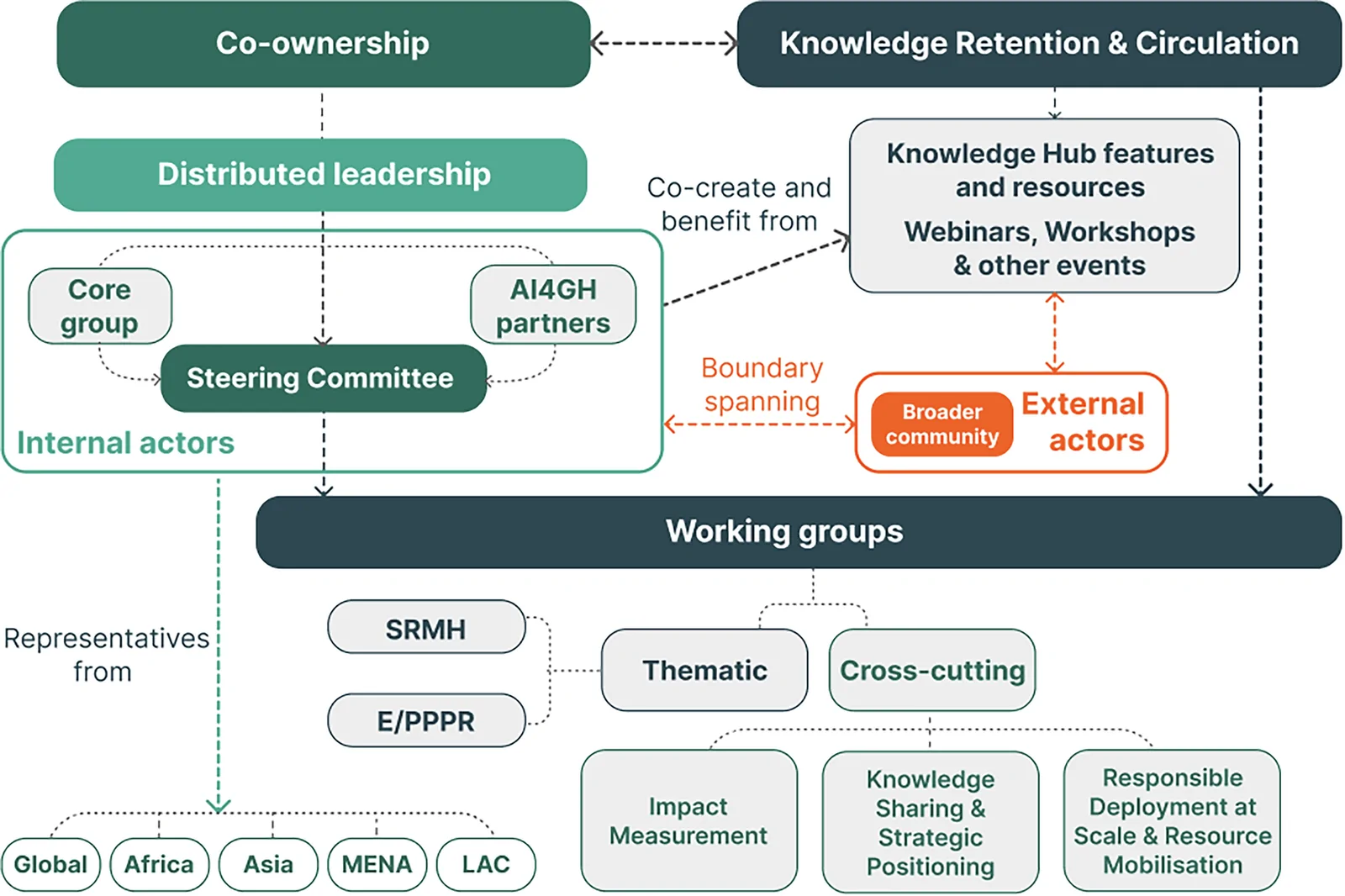
AI4GH CoP organisational structure. AI4GH Artificial Intelligence for Global Health, E/PPPR Epidemic/Pandemic Prevention, Preparedness and Response, LAC Latin America and the Caribbean, MENA Middle East and North Africa, SRMH Sexual, Reproductive and Maternal Health.

Moving to the facets convening and circulation/collaboration, we identified multiple activities where both internal and external stakeholders engage around community vision, as listed in Table 1. Opportunities for convening include in-person and online interactions through strategic meetings among partners (Table 2), as well as thematic and cross-cutting working groups (WGs). These WGs provide a structured environment for focused discussions, enhancing collaboration and co-creation among partners. Figure 3 illustrates how the ‘Knowledge retention and circulation’ and ‘Co-ownership’ domains interconnect in the CoP’s organisational structure.

Working groups emerged not only as convening mechanisms but also as components of the CoP’s governance structure. Two thematic WGs focus on SRMH and E/PPPR, respectively. Cross-cutting WGs were created to address shared challenges, including measuring the impact of AI4GH projects, strengthening knowledge-sharing practices, strategic positioning, responsible deployment at scale and resource mobilisation (Fig. 3). These groups engage in regular discussions and project updates to consolidate best practices, identify opportunities for collective progress and produce collaborative learning materials.

Workshops and webinars, delivered in hybrid, online, or in-person formats, further support convening and capacity-building for AI4GH grantees, subgrantees and the broader community, bringing together researchers, practitioners, policymakers and civil society. Priority topics for these events were identified through discussions with grantees. The full list of workshop and webinar topics is available online^22^.

The AI4GH Knowledge Hub, available in English, French and Spanish, serves as a central platform for knowledge sharing and collaboration across regions. It hosts curated resources, selected based on scoping activities through discussions with partners and gap analysis. It also features a dedicated subpage for Responsible AI concepts and materials. Additionally, the hub benefits from being hosted within The Global Health Network platform, which provides access to additional global health research toolkits. The Hub highlights ongoing projects and integrates features such as the Connect and Collaborate Tool, a shared events calendar and a newsletter. Alongside presence at global conferences, these mechanisms extend the reach of outputs, support joint initiatives and strengthen knowledge circulation within and beyond AI4GH-funded projects.

### Community manager’s role: community management, user experience and measurement

Community management is led by a core team within The Global Health Network, comprising a project manager, a Monitoring, Evaluation and Learning (MEL) manager and regional coordinators for Asia, Africa and LAC. The team focuses on enhancing user experience through platform optimisation for low-bandwidth environments, lightweight downloadable materials, offline and multilingual content and accessible navigation. Following partner meetings and events, The Global Health Network gathers feedback through forms and sessions to inform improvements for future activities.

**Fig. 4:**
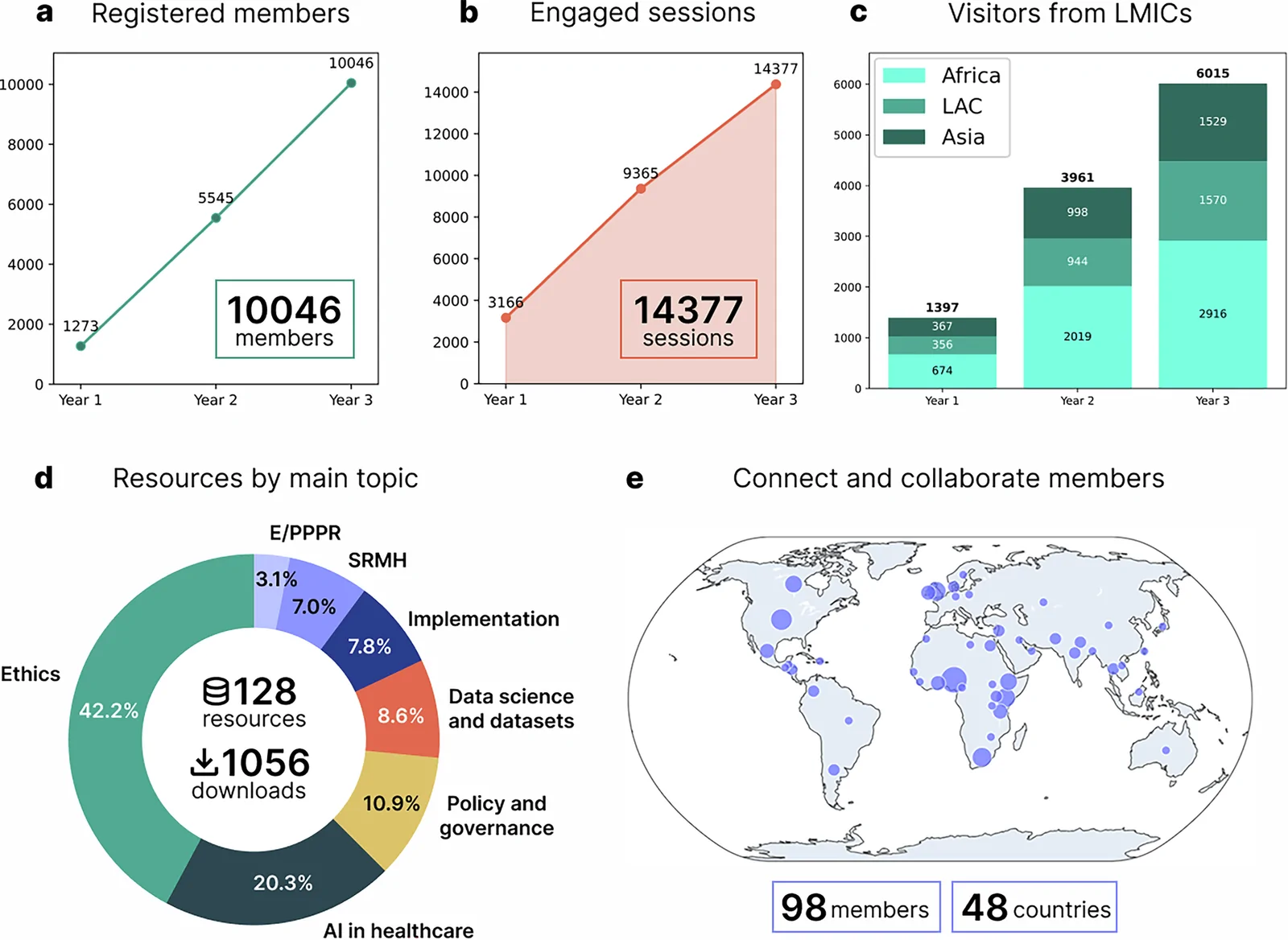
AI4GH CoP knowledge hub metrics. **a** Registered members per year (cumulative to the end of September 2025). **b** Engaged sessions per year (cumulative to the end of September 2025). An engaged session is defined as a session lasting longer than 10 s, including a conversion event, or containing at least two pageviews or screenviews. **c** Visitors from LMICs per year (cumulative to the end of September 2025). **d** Resources by main topic. **e** ‘Connect and Collaborate’ members (September 2025). AI Artificial Intelligence, E/PPPR Epidemic/Pandemic Prevention, Preparedness and Response, LMICs Low- and middle-income countries, SMRH Sexual, Reproductive and Maternal Health.

Regarding measurement, the knowledge hub currently has 10,046 registered members and 14,377 cumulative engaged sessions over three years, with strong engagement across LMICs, particularly in Africa. The resource library contains 128 materials, downloaded 1056 times, including articles, guidelines, frameworks, workshop recordings, websites, datasets, e-learning resources, lectures, podcasts, policy briefs, technical reports and panel discussions. They are focused mainly on AI ethics (42.2%) and AI applications in healthcare (20.3%), with additional materials on policy, governance, data science, implementation and AI for SRMH and E/PPPR. The ‘connect and collaborate’ feature allows users to add themselves to a global map, identify peers by research area, skills or location and establish collaborations. At present, 98 collaborators are listed across 48 countries (Fig. 4).

### Overall assessment

**Fig. 5:**
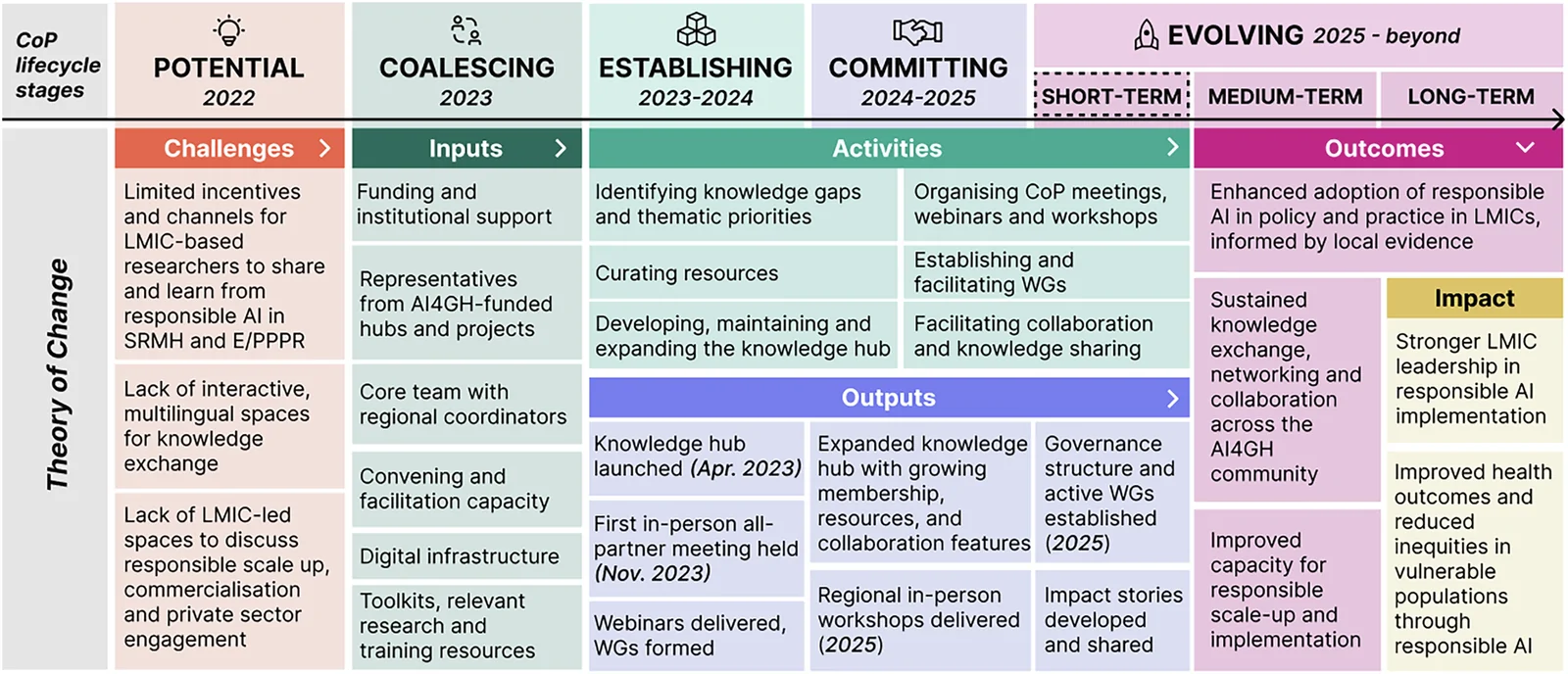
Theory of change and development timeline of the AI4GH CoP. The horizontal arrow represents the CoP’s progression from the Potential to the Evolving stage. Each column aligns a stage with the theory of change element introduced at that point; arrowheads indicate that each element persists through the subsequent stages. The dashed rectangle marks the current stage (Evolving, short-term). Dates in brackets indicate milestones. AI Artificial Intelligence, CoP Community of Practice, E/PPPR Epidemic/Pandemic Prevention, Preparedness and Response, LMICs Low- and middle-income countries, SMRH Sexual, Reproductive and Maternal Health, WGs Working groups.

Through the community assessment, we positioned the AI4GH Community of Practice in the early evolving stage, according to the CoP lifecycle proposed by Wenger^23^. At this stage, members assume stewardship of the CoP domain (AI for global health) and focus on translating outputs into pathways that lead to outcomes, aligning with the initiative’s theory of change. Figure 5 situates the CoP’s current stage within the broader implementation pathway from challenges to impact.

We identified signs of maturity, such as distributed leadership, an engaged core group and a growing repertoire of resources and activities. However, we also detected gaps in sustaining learning loops and linking outputs more systematically to practice and policy. So far, monitoring has focused on short-term indicators such as hub visits, shared resource and meetings held, which capture activity but do not measure professionals impacted or changes in practice and policy. From year five onwards, the community plans to assess these medium- and long-term outcomes, using focus group discussions and interviews.

In addition to the internal learning outputs, we identified lessons potentially transferable to other AI-for-health ecosystems. The first is the value of WGs in recognising and addressing shared challenges. For example, the SRMH WG highlighted difficulties in engaging governments to integrate AI into national health systems, prompting additional workshops and guidance for subgrantees. The cross-cutting WGs also revealed other common barriers, such as limited national and regional AI strategies, data availability, technical capacity, transitioning AI interventions from research to sustainable applications and assessing project impact. These needs shaped priority topics for resources and capacity-building events, enabling activities that support continuous learning and practical application tailored to the community context.

A further transferable lesson emerged from the workshops. They provide a structured forum for engaging critical stakeholders and addressing region-specific priorities. One example is the 2025 in-person workshop in the MENA region, where participants highlighted the urgent need for national and regional AI governance and regulation and agreed to map and advance this landscape as a next step. In Africa, a workshop explored the practical applications of generative AI, particularly chatbots, to enhance healthcare delivery. Each event involved government representatives, which helps to translate workshop discussions into policy and regulatory action.

## Discussion

This case study aimed to describe and reflect on the status, activities and outputs of the AI4GH CoP. The analysis, structured around the eight facets of the Community Roadmap, revealed a CoP with strong co-ownership, multiple mechanisms for knowledge retention and circulation and a clear understanding of the community manager’s role. These features have amplified the collective impact of AI4GH investments within and beyond the funded projects.

Through the community assessment, we identified features of the CoP model that help bridge gaps between AI research, implementation and scaling in LMICs. In their 2002 book^9^ on cultivating CoPs, Wenger and colleagues noted that in fast-moving fields, people often form communities to keep pace with rapid technological change. More than two decades later, this pattern is also evident in organisations working in AI for global health. They operate in an environment characterised by interdisciplinarity and rapid change. Although information is widely accessible, it does not necessarily translate into practical results, given the difficulty of identifying credible and contextually appropriate knowledge sources. In this context, a CoP enables practitioners to set their learning priorities, assume responsibility for the knowledge they require and link learning with engagement, professional development and the strengthening of organisational capabilities.

An example of this dynamic is the way the AI4GH CoP has evolved to align with WHO priorities for AI governance^24^, specifically in the areas of ethics, regulation, implementation and operation. Ethics is prominently addressed through the majority of curated materials in the knowledge hub. Additionally, member needs prompted the launch of a dedicated Responsible AI page within the hub, reflecting engagement and commitment to integrating health equity into the AI lifecycle^25^, consistent with the CoP’s purpose. Challenges related to regulation, implementation and operation are primarily addressed through cross-cutting WGs and multistakeholder events, including participation from government representatives. Members shaping the learning agenda also contributed to the development of a WG focused on responsible deployment at scale and resource mobilisation. Working closely with experts in AI governance and health systems, the group aims to support ethical, equitable and sustainable scaling of AI innovations during the transition from proof-of-concept to scale phase, across both for-profit and not-for-profit models. The group is also examining opportunities to coordinate funding strategies across initiatives.

Because CoPs are not constrained by formal structures, they can foster connections across organisational and geographic boundaries^9,23^. Knowledge and practices developed in one region can inform solutions elsewhere, creating a living repository that supports scaling, standardisation and coordination of related initiatives. For example, a solution developed by a team in South America could address a challenge faced by a team in North Africa.

Regarding the collaborating and convening format, in the AI4GH CoP, WGs, webinars, workshops and the digital knowledge hub emerged as central structures for fostering knowledge sharing, capacity building, collaboration, co-creation and accelerating research. Similar outcomes have been observed in other projects within The Global Health Network^12,26^ and in other global health CoPs^27–30^. These mechanisms align with the framework proposed by Frehywot and Vovides^17^, which identifies technical working–learning groups, a central digital hub, webinars and workshops as core components for stakeholder engagement and CoP sustainability in the context of AI in global health workforce training.

However, learning in a community of practice is not a transfer of information from an expert to a novice. It is a continuous process in which members generate ideas, test them in practice and refine them through reflection, creating ongoing learning loops^9^. The sustainability of a CoP depends on the value members find in these interactions. In the AI4GH CoP, mechanisms such as distributed leadership, WGs, workshops and durable artefacts like the Knowledge Hub appear to drive meaningful interaction and support sustainability. Determining which elements are perceived as most valuable requires in-depth assessments with members and differentiating intrinsic individual motivation from organisational expectations. Further CoP research is also needed to examine how partners, projects, impacted communities and the wider AI-for-health ecosystem interact within the CoP and influence its functioning across LMICs.

As Hosny and Aerts already emphasised five years ago, AI interventions in LMICs should be initiated, owned and administered by local stakeholders^4^. Despite contextual differences, LMICs face common challenges that can be addressed through collaborative learning^1^. In line with SDG 17, ‘Partnerships for the Goals,’ a CoP can consolidate fragmented initiatives, bridge capacity gaps and facilitate knowledge sharing. The AI4GH CoP exemplifies how Global South leadership contributes to decolonising global health research by amplifying local expertise and shifting the locus of power in AI development and governance. However, achieving the initiative’s full potential requires moving beyond reliance on individual contributions or funding cycles, highlighting the need for system-level changes.

Investing in digital infrastructure is a critical factor. Many public health institutions rely on outdated systems that limit large-scale AI-driven analysis^16^. Several countries also lack AI regulations, secure data-sharing strategies and policies governing big data. Ethical safeguards, data governance, privacy, consent, cybersecurity and explainability are recurring themes in CoP discussions, reflecting priority areas for policy agendas^24^. Furthermore, training and capacity building require sustained investment. Although resources are available, the rapid evolution of AI demands continuous education. There is a need for institutions to integrate AI education within strategic planning to ensure staff are prepared to work within relevant legal and regulatory frameworks and apply responsible AI practices.

Furthermore, ensuring equity and inclusion is fundamental to the success of AI projects in global health. Monitoring and evaluating the representativeness of datasets and the level of community involvement can provide insight into the relevance of these technologies. Measuring impacts on target groups, end-users, health workers, health outcomes, equity, scalability, sustainability, health system adoption and policy implementation are essential to evaluate the ability of AI interventions to respond effectively to local needs. This can also support cross-border validation of AI models. By integrating these dimensions, AI initiatives can move beyond technical innovation to contribute to equitable health outcomes.

In this case study, we describe the current AI4GH CoP structure, activities and outputs while reflecting on its performance. The AI4GH CoP leverages digital infrastructure, a distributed leadership model and working groups to foster knowledge sharing and collaboration to address health priorities in LMICs. By integrating inclusive governance, local capacity-building and cross-regional collaboration, a CoP can accelerate responsible AI research in global health. It also provides a pathway for strengthening equitable AI research ecosystems and bridging health inequalities and North–South divides. These structural and operational elements set the stage for understanding the CoP’s functioning and the lessons it offers for similar initiatives.

Communities of Practice can take many forms, but all share three core elements: a domain, a community and a practice. The AI4GH CoP exemplifies a large, distributed, heterogeneous and intentional global community, demonstrating how CoPs can support multidisciplinary, fast-evolving fields. One transferable lesson from this study is that establishing a global CoP requires intentional design to ensure equitable regional representation and broad participation. Region-specific engagement strategies and multilingual materials help secure inclusive representation and participation. Our analysis also highlights that global CoPs rely not only on strong community management but also on members actively shaping their learning according to practical needs. Access to digital resources, such as the Knowledge Hub, is insufficient without active facilitation to translate resources into learning and collaboration. Diverse convening and collaboration mechanisms support this facilitation, strengthening knowledge exchange. Consequently, the CoP functions as a strategic bridge between networks and knowledge centres.

Maintaining and expanding this impact depends on ongoing monitoring, assessing effects on policy and practice and cultivating enduring partnerships that reinforce local leadership, sustainability and boundary-spanning. These efforts can shape discourse, policies and innovations across the Global South.

This case study has important limitations. The reporting approach captured the perspectives of a limited subset of the CoP. To minimise bias, analysis and reporting were conducted collaboratively and findings were cross-checked against documented activities and outputs. However, broader participation, including members, core groups and stakeholder surveys, would have strengthened the study and will be considered in future research. Accordingly, this work should be viewed as a foundational reference for a high-level understanding of the CoP’s structure and development. Not including interviews or surveys in this inaugural description of AI4GH CoP was intentional. Our aim at this stage was to establish a structured account of the initiative’s early development and characterise developmental processes before undertaking more resource-intensive assessments of members’ experiences.

Future CoP research will extend this groundwork through participatory methods, such as in-depth interviews and focus groups, to capture testimonies, explore synergies, members’ experiences and participation. Ongoing efforts, such as the Impact Stories project (Table 1, question 27), will further inform this assessment. The application of the Community Roadmap in this case study may equip other groups with a practical model for initiating, structuring or refining their own CoPs. This work can also serve as a ‘boundary object’, in Wenger’s sense^31^, supporting connections across different practices.

## Methods

### Study design and framework

We conducted an intrinsic descriptive case study^32^ using an interpretative approach^33^. This design enabled an in-depth exploration of the AI4GH CoP within its real-life context^32^. The primary objective was to describe the status, activities and outputs of the CoP. The secondary objectives were: (i) to assess the CoP’s functioning using a structured instrument, identifying strengths, gaps and contextual influences; and (ii) to generate insights that could inform the CoP’s ongoing development and inform similar initiatives in LMIC contexts.

We employed the Community Roadmap tool developed by the European Commission as a framework to guide the report and assess the CoP’s functioning^20^. This tool, created through a multi-step methodology^20^, was designed to evaluate a community’s current state and identify areas for improvement. It comprises 27 questions and organises analysis around eight facets of the Communities of Practice Success Wheel: Vision, Governance, Leadership, Convening, Collaboration/Cooperation, Community Management, User Experience and Measurement. The framework helps CoPs translate their strategic vision into an actionable overview that can enhance their operational model and member experience. The questions were used as presented in the tool.

This tool was selected because: (i) it is a validated methodology for supporting the design thinking process within CoPs as defined by Wenger^9^; (ii) it was developed for large-scale, multi-stakeholder CoPs similar to AI4GH; and (iii) it facilitates describing a community’s journey, evaluating performance and communicating value both internally and externally. This framework was designed to support understanding the community’s current status, identify opportunities and areas for improvement and guide the process of mapping CoP stakeholders, structures and initial functioning. Our expectation is that the structured roadmap will provide direction for future, more detailed evaluations, including members’ experiences.

### Data collection and analysis

We collected data from multiple document types, including all formal materials generated during the CoP’s establishment and development, from its inception in 2023 to September 2025. Documentary sources were included if they (i) were produced by AI4GH CoP members; (ii) directly informed operational, governance or strategic development; and (iii) were formally circulated as part of the initiative’s documentation workflow. Therefore, eligible materials comprised the initial CoP proposal, the theory of change, technical reports, operational plans, communication and engagement strategies and routine activity summaries. Aggregated website metrics from the AI4GH Knowledge Hub were retrieved on 30 September 2025 using Google Analytics 4 (GA4) as an indicator of platform usage.

Using these sources, the Community Roadmap questions^20^ were first answered by a new CoP member (M.M.G.A) who was previously unfamiliar with the CoP’s processes and materials. Other core members (M.R. and M.B.) then independently reviewed the responses and provided feedback, which was incorporated through iterative refinements. This approach was intended to reduce ‘curse of knowledge’ bias^34^ in the synthesis by incorporating the fresh perspective of a new member^35^, less influenced by prior knowledge or involvement. Long-standing core members and partner representatives supported the interpretation of the findings and engaged in rounds of review to ensure accuracy and consistency with the CoP’s context.

### Ethical considerations

This case study used only documentary sources and aggregated website metrics. No surveys or interviews with stakeholders were conducted or analysed. No personal or sensitive data were collected, stored, or processed. Patients and members of the wider community were not involved. Group discussions held for the roadmap application were not transcribed, registered, or subjected to further analysis. On this basis, ethical approval was not required.

## Data Availability

The data underlying this article were derived from project documentation, internal reports, website analytics and operational knowledge gained during the design and implementation of the AI4GH Community of Practice. As these materials include internal and partner-owned information, they are not publicly accessible. Data can be made available upon reasonable request to the corresponding author and are subject to permission from the partner organisations.
